# Thyroid hormone resistance and the value of genetics: Three case reports

**DOI:** 10.1097/MD.0000000000014675

**Published:** 2019-03-01

**Authors:** Xiao Xiao, Chen Lv, Tianxiao Zhu, Huiling Chen

**Affiliations:** a Department of Endocrinology, Xiangya Hospital, Changsha, Central South University; b School of Nursing, Xiangnan University, Chenzhou; c Department of Orthopedics, Xiangnan University Affiliated Hospital, Chenzhou; d Electrocardiogram Room, Hunan Provincial Maternal and Child Health Hospital, Chenzhou, Hunan, China.

**Keywords:** case report, gene mutation, somatostatin suppression test, thyroid hormone resistance (RTH)

## Abstract

**Rational:**

Thyroid hormone resistance (RTH) is a rare disease that is characterised by a lowered sensitivity of the target organs to thyroid hormone. Herein, we present 3 cases of confirmed RTH, with the support of clinical lab results and/or gene sequencing at diagnosis.

**Patient concerns:**

The 3 included patients were found to have elevated levels of free T_3_ (FT_3_), free T_4_ (FT_4_), and non-supressed levels of thyroid stimulating hormone (TSH).

**Diagnosis:**

All patients were tested for thyroid antibodies, somatostatin suppression, vision and hearing at diagnosis. Electrocardiography (ECG), thyroid ultrasonography, and magnet resonance imaging (MRI) of the sellar region were also performed. Furthermore, gene sequencing was used to detect the thyroid hormone receptor beta (THRB) gene mutation.

**Interventions:**

Patient treatment was individualised. Patients were given levothyroxine sodium or a low dose of thyroiodin, depending on the individual symptoms.

**Outcomes:**

After treatment, thyroid function was stable in 2 of the teenage patients. No evidence of worsening thyrotoxicosis was observed.

**Lessons:**

Gene sequencing should be considered together with clinical lab results, including somatostatin suppression testing, before approaching a diagnosis of RTH.

## 1 Introduction

The syndrome of thyroid hormone resistance (RTH) is a rare condition of the endocrine system in which the sensitivity of the target tissues to thyroid hormone (TH) is decreased. RTH is typically characterised by an increased level of serum free T_3_ (FT_3_), free T_4_ (FT_4_), and a non-suppressed level of thyroid stimulating hormone (TSH).^[1]^ Since the 1st patient with RTH was identified by Refetof in 1967,^[2]^ more than 3000 cases of this condition have been reported.^[3]^ Among these cases, more than 300 pedigrees have been reported in previous studies.^[4]^ The morbidity of RTH is about 1 in 40,000.^[5]^

The main molecular mechanism of RTH is a mutation of the thyroid hormone receptor beta (THRB) gene, which encodes the TH receptor β (THRβ).^[6]^ Currently, more than 100 mutational sites have been reported, most of which are located in 3 “hotspot” regions.^[4]^ These carboxyl terminal ligand binding regions of THRβ encoded by exons 7 to 10 in the THRB gene are amino acids 234 to 282, 310 to 353, and 429 to 46.^[7,8,9,10]^

The clinical manifestations of RTH vary between individuals and some cases can even be asymptomatic. Common symptoms observed in patients with RTH include goiter, thyrotoxicosis, colour blindness, amblyopia, dysacusis, somatic defects (e.g. bird-like face, craniosynostosis) and central nervous system (CNS) damage (e.g. hypophrenia, expressive dysphasia).^[9,12,13]^ The diagnosis of RTH is, therefore, difficult, due to inconsistencies in the clinical manifestations and the lack of diagnostic guidelines. Detailed investigations, considering clinical manifestations, lab results and necessary radiology tests, should be conducted before a diagnosis of RTH is made.^[11,14]^ Alternatively, gene sequencing provides a much easier method of diagnosis.^[11]^ Here, we present 3 cases of RTH to demonstrate the use of gene testing during diagnosis.

## 2 Methods

### 2.1 Ethical approval and consent for publication

The present study was approved by the Human Research Ethics Committee of Xiangnan University Affiliated Hospital. Consent forms for publication of the cases were obtained from the patients.

### 2.2 Somatostatin suppression test

Octreotide suppression was tested in all 3 of the included cases. Patients were given 3 subcutaneous injections of octreotide at 8 hours intervals (100 μg per injection).^[15]^ Peripheral blood samples were collected at time 0 (before the 1st subcutaneous injections of octreotide), followed by collections at the following times from time 0: 2 hour, 4 hour, 6 hour, 8 hour, and 24 hour. At each time point the levels of FT_3_, FT_4_, and TSH were tested. Serum FT_3_, FT_4_, and TSH levels were detected using chemiluminescence immunoassay.^[16]^

### 2.3 Molecular test

#### 2.3.1 Extraction of genomic DNA from peripheral blood

The extraction of genomic DNA from peripheral blood samples in these 3 cases was performed following the instructions of the DNA extraction kit (SQ Blood DNA Kit II D0714–50, OMEGA Bio-tek Inc, GA).

#### 2.3.2 Amplification of the THRB gene

Amplification of the exons 7 to 10 of the THRB gene was performed using each patient's DNA as a template. Primers were designed and synthesised by Sangon Biotech (Shanghai) Co, LTD, China (See Table 1). Polymerase chain reaction (PCR) was performed according to the instructions of the Taq PCR Master Mix Kit (KT201; Tiangen Biotech Co, Ltd, Beijing, China). The PCR products were preserved at 4°C.

**Table 1:**
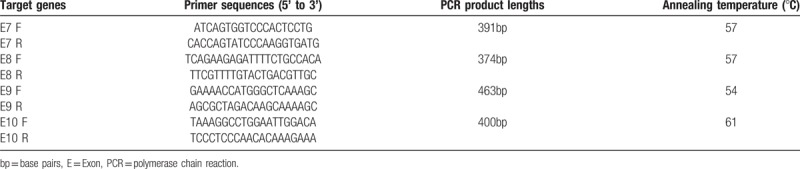
Primer sequences and polymerase chain reaction product lengths of thyroid hormone receptor beta gene exon 7 to 10.

#### 2.3.3 Electrophoresis of the PCR products

The PCR products were electrophoresed on a 2% agarose gel for 30 min and the results were analysed by a gel imaging analysis system (BIO-PRO CN-UV/WL, SIM International Co, LTD, CA).

#### 2.3.4 Sequencing of the THRB gene

The PCR products were purified and sequenced bidirectionally by Sangon Biotech (Shanghai) Co, LTD, China. The gene sequencing results were compared using the THRB gene exons 7 to 10 on Genbank (www.ncbi.nlm.nih.gov/genbank/).

## 3 Case reports

### 3.1 Case 1

A 14-year-old girl was referred to our hospital, due to suspected hyperthyroidism, in October 2012. The parents patient had a reported history of being irritable and overeating, according to her. She performed poorly at school and had experienced grade retention twice. Her communication ability was not at the same level as other children of her age. The local hospital diagnosed her with Graves’ disease and prescribed anti-thyroid treatment before she was referred.

At presentation, the patient was 150 cm in height and weighed 44 kg. These measurements were within the normal range for her age. Her heart rate was 112 bpm (beats per minute). A physical examination revealed a 2nd degree of thyroid enlargement. No craniofacial deformity was observed.

Following admission, the results of thyroid function tests indicated that the patient had elevated FT_3_ (11.02 pmol/L), FT_4_ (36.11 pmol/L), and TSH (4.32 μU/mL) levels. The levels of hyroid peroxidase antibodies (TPOAb), thyroglobulin antibodies (TgAb), thyrotrophin receptor antibodies (TRAb), and sex hormone-binding globulin (SHBG) were within the normal range. Tests of visual acuity indicated the presence of amblyopia. The results of a colour vision test and hearing tests were normal. Electrocardiography (ECG) revealed sinus tachycardia. No pituitary tumour was visualised through magnetic resonance imaging (MRI). Thyroid ultrasonography revealed diffuse enlargement of the thyroid glands.

**Figure 1:**
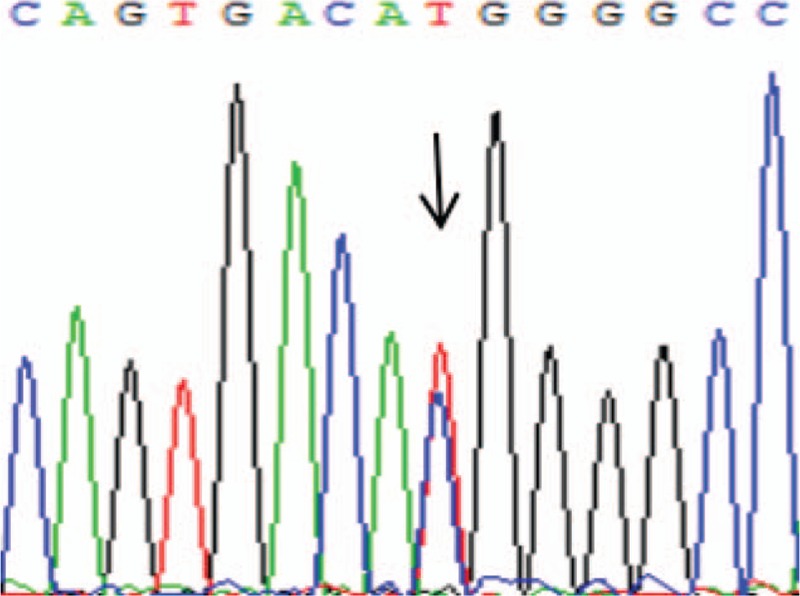
Gene sequencing result of THRB gene exon 9 in case 1. THRB = thyroid hormone receptor beta gene.

Based on the aforementioned investigations, it was suspected that the patient had RTH. The patient was then sent for a somatostatin suppression test and “hot spot” gene sequencing. The somatostatin suppression test revealed that the levels of serum FT_3_ and TSH were suppressed by less than 30% (see Table 2). A heterozygotic mutation was detected at R338W of exon 9 in the THRB gene (see Fig. 1).

**Table 2:**
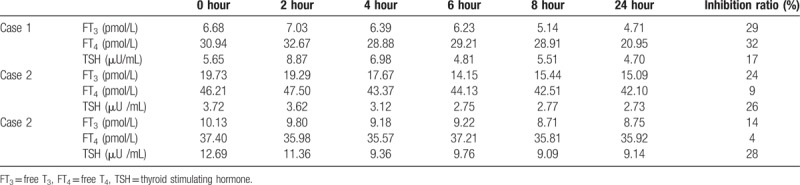
Results of somatostatin suppression tests.

As a result, the patient was prescribed Levothyroxine sodium tablets, 25 μg, to be taken once per day. Unfortunately, after 12 months of treatment, the symptoms of irritability and overeating were not improved. There was no evidence of a decrease in the thyroid enlargement. Thyroid function tests revealed that the levels of FT_3_ (14.31 pmol/L), FT_4_ (42.74 pmol/L), and TSH (4.05 μU/mL) remained elevated. The patient is still undergoing follow-up evaluation.

### 3.2 Case 2

A 12-year-old girl was referred to our hospital from her local hospital in July 2014. An enlarged lump on the patient's neck was identified by her mother, causing her to seek medical help at the local hospital. The patient's grades were within the average level at school, with a relatively poor communication ability compared to that of her peers. The local hospital diagnosed her with Graves’ disease and put her on anti-thyroid treatment.

At presentation, the patient was 148 cm in height and weighed 42 kg. Her heart rate was 107 bpm. Physical examination revealed a 2nd degree of thyroid enlargement. Her facial structure was classed as being bird-like.

After admission, the endocrine lab results revealed increased FT_3_ (15.65 pmol/L) and FT_4_ (52.50 pmol/L) levels. However, the levels of TSH were within the normal range (3.34 μU/mL). The levels of TPOAb, TgAb, TRAb, and SHBG were also within the normal range. Tests for visual acuity, colour vision, and hearing were unremarkable. The ECG showed tachycardia. The sellar MRI came back negative. Ultrasonography indicated diffusely enlarged thyroid.

**Figure 2:**
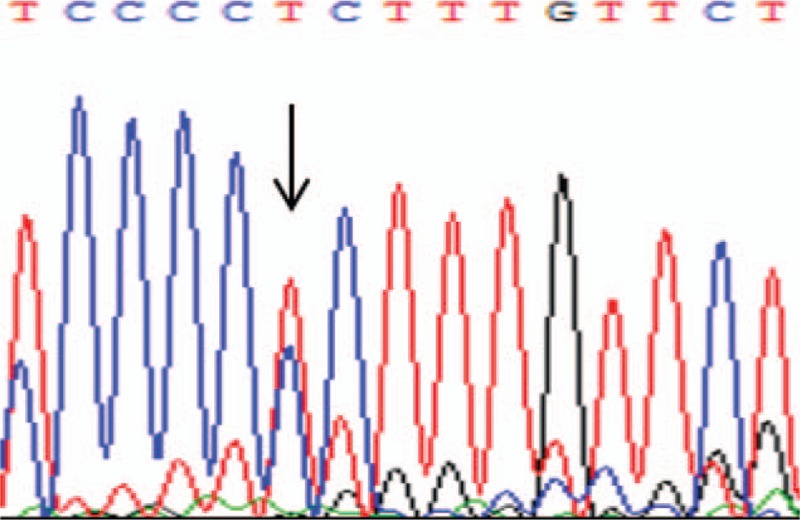
Gene sequencing result of THRB gene exon 10 in case 2. THRB = thyroid hormone receptor beta gene.

The patient then underwent a somatostatin suppression test and gene sequencing for diagnosis. The somatostatin suppression test revealed that the serum FT_3_, FT_4_, and TSH levels were suppressed by less than 30% (see Table 2). The results of gene sequencing revealed a heterozygotic mutation, P453S of exon 10 in the THRB gene (see Fig. 2).

Thyroiodin, at a dose of 40 mg per day, was prescribed to the patient. After 3 months, thyroid function tests revealed that the levels of FT_3_ (9.67 pmol/L) and FT_4_ (26.10 pmol/L) had improved but the TSH level had worsened (18.06 μU/mL). The dose of thyroiodin was then lowered to 20 mg per day. At the 3-month follow-up, thyroid function tests showed the following results: FT_3,_ 15.86 pmol/L; FT_4,_ 34.60 pmol/L; and TSH, 7.19 μU/mL. The dose was further reduced to 10 mg per day. After a further 3 months, the thyroid function tests showed improved (FT_3_, 12.93 pmol/L; FT_4,_ 48.93 pmol/L; and TSH, 1.95 μU/mL) compared to the initial results. There was no evidence of hyperthyroidism symptoms. However, the condition of the enlarged thyroid condition was not significantly altered.

### 3.3 Case 3

A 34-year-old female was referred to the neurosurgical department of our hospital due to the discovery of a pituitary lesion following an accidental trauma in September 2014. Thyroid tests revealed elevated levels of FT_3_ and FT_4_ with non-supressed TSH expression. The clinical symptoms gave no evidence of thyrotoxicosis. The MRI results indicated an enlarged hypophyseal fossa, and a cystic-solid lesion, 2.7 cm × 1.7 cm × 1.7 cm in size, located in the sella region. Therefore, the patient underwent surgical resection of the lesion. The post-operative pathological report indicated pituitary adenoma, TSH (-). The patient then visited our endocrine outpatient clinic for further consultation.

At presentation, the patient complained of a history of irregular menstruation for approximately 15 years, with the cycle length of 1 to 4 months. Her heart rate was 72 bpm. Physical examination revealed a 1st degree of thyroid enlargement. No other remarkable medical condition was observed.

Thyroid function tests showed elevated FT_3_ (7.78 pmol/L), FT_4_ (31.00 pmol/L), and TSH (13.04 μU/mL) levels. The TPOAb, TgAb, (TRAb, and SHBG levels were within the normal range. Tests for visual acuity, colour vision and hearing all showed results within the normal range. The ECG showed a normal sinus rhythm. Ultrasonography revealed enlarged thyroid glands.

For diagnosis, a somatostatin suppression test and gene sequencing were performed. The results of the somatostatin suppression test showed that the serum levels of FT_3_, FT_4_, and TSH were suppressed by less than 30% (see Table 2). The results of gene sequencing were negative for any mutation.

Because there was no obvious discomfort, the patient refused any further treatment.

## 4 Discussion

In this article, we presented 3 cases of RTH that were confirmed through clinical lab results and gene sequencing. In these cases, increased serum FT_3_ and FT_4_ levels, together with non-suppressed TSH, were observed. All of the cases presented with goiters at different degrees. Furthermore, the patient in case 1 had thyrotoxicosis. No abnormalities were found in the levels of pituitary hormones, estrogen or progesterone. However, the patient in case 3 suffered irregular menstruation for approximately 15 years. Overall, the clinical manifestations of RTH in these cases were consistent with the previous literature.^[7,11]^

The clinical manifestations of RTH are diverse between patients. Individuals with the same mutation site may present with different clinical manifestations, even in 1 pedigree.^[17]^ The most common symptom of RTH is enlarged thyroid glands, goiter,^[18]^ tachycardia, mental retardation, hyperkinetic behaviour, achromatopsia, short stature (< 5%), and facial dysmorphia (e.g. bird-like face, pigeon breast) as well as other types of body dysmorphia.^[7,11]^ Therefore, the presence of the aforementioned symptoms may support a diagnosis of RTH, especially in juveniles.

Serum levels of FT_3_, FT_4_, and TSH are the primary screening tests for RTH. Other biochemical tests, including levels of α-TSH, SHBG, and thyroid antibodies, as well as radiologic assessment of the sellar region through MRI are used for further diagnosis and differential diagnosis.^[7,11,19,20]^ In some cases, RTH needs to be distinguished from thyroid autoimmune diseases and pituitary TSH-secreting adenoma (TSHoma). In TSHoma patients, the levels of α-TSH and SHBG are usually significantly elevated. Patients with TSHoma often present with synchronous increases in FT_3_, FT_4_, and TSH levels. A somatostatin suppression test can also be used of differentiate between a diagnosis of RTH and that of TSHoma.^[15,21]^ In general, the inhibition rate of TSH was over 30% in cases of TSHoma, while, in cases of RTH, the inhibition rate of TSH was less than 30%.^[15]^ Moreover, sellar MRI can be used to detect any pituitary lesions, which is an essential method for the differentiation between RTH and TSHoma.

Through gene sequencing, in these 3 cases, 2 patients were found to have genetic mutations at sites that have been reported to be involved with RTH.^[22,23]^ According to the literature, the clinical manifestation is not closely linked to a specific mutation point.^[14]^ However, in homozygotes, the symptoms appear to be more severe. This may be affected by the expression level of the defective receptors.^[18]^

In cases 1 and 2, the clinical symptoms, thyroid lab results, MRI and somatostatin test results all lead to a diagnosis of RTH. Genetic sequencing for these 2 patients revealed mutations, confirming the clinical diagnosis. However, in case 3, no mutation was found in the “hotspot” region and no symptoms were observed. However, initially this patient had a lesion in the sellar region and underwent neurosurgery. The pathological report and immunohistochemical analysis in this case did support a diagnosis of RTH.

Gene mutation can be added to the diagnostic guidelines as a supporting criterion for RTH.^[11]^ However, no mutations are observed in 15% of reported patients.^[11]^ This may be because exon mutations in the non-hotspot regions of the THRB gene or THRA gene,^[3,24–26]^ problem of ligand^[27]^ and thyroid hormone transporter,^[28,29]^ defective backward acceptor^[7]^ were not included. Recent studies have suggested that the presence or absence of a mutation in the THRB gene has no effect on the patient's clinical performance, biochemical examination or dynamic test.^[14,18]^ At present, the mutation sites of RTH are still not fully understood. Therefore, it is necessary to consider the genetic outcome together with clinical results before making a diagnosis.

Unfortunately, there is currently no approach to fully cure RTH. According to the literature, the most effective treatment is 3,5,3,’-triiodothyroacetic acid (TRIAC),^[30]^ which can effectively inhibit TSH and TH levels and possibly reduce thyroid gland swelling. D-T_4_ (dextrothyroxin) can be offered as a choice for RTH patients with hyperthyroidism, as TRIAC is not effective in the treatment of such cases. Conversely, L-T_3_ (levotriiodothyronine) treatment can used for patients with hypothyroidism.^[31]^ Anti-thyroid treatment is usually not recommended for patients with RTH, as it may cause malignant circulation and may even induce the development of pituitary tumours. Moreover, individualised therapy is required because of the variety of clinical features in these patients. Unfortunately, TRIAC, D-T_4_ and L-T_3_ are currently not available at our hospital. Patient 1 was prescribed levothyroxine tablets, however, there was no significant change in the levels of TSH, FT_3_ or FT_4_ after 12 months of follow-up. In case 2, the patient was treated with thyroiodin. The response to various doses of the drug indicated that a lower dose, of 10 mg per day, reduced TSH levels by about 40%. However, but no significant change in the levels of FT_3_ or FT_4_ were observed at this dose. After treatment, no symptoms of thyrotoxicosis were observed in case 1 and the status of patient 2 remained stable.

In conclusion, we presented 3 cases of RTH that were diagnosed in our hospital. We introduced gene sequencing, together with clinical investigations, in the diagnosis of these patients. In cases which present elevated FT_3_ and FT_4_ levels and non-suppressed TSH levels, together with suspected RTH clinical symptoms, gene sequencing should be encouraged, to confirm a diagnosis of RTH. In patients with RTH-like thyroid function changes but no genomic mutations, RTH should still be highly suspected.

## Acknowledgments

The authors would like to thank all the staff at the Department of Endocrinology at Xiangya Hospital.

## Author contributions

**Data curation:** Chen Lv, Tianxiao Zhu.

**Formal analysis:** Chen Lv.

**Investigation:** xiao xiao.

**Methodology:** xiao xiao.

**Resources:** Huiling Chen.

**Software:** Huiling Chen.

**Supervision:** Huiling Chen.

**Writing – original draft:** xiao xiao.

**Writing – review & editing:** xiao xiao.
